# Supplementary material for: Morphology Determines an Efficient Coherent Electron Transport for Push–Pull Organic Semiconductors Based on Triphenylamine and Dicyanovinyl Groups
Source: Materials (Basel). 2023 Mar 18;16(6):2442. doi: 10.3390/ma16062442 (PMC10058145; doi:10.3390/ma16062442)
Supplement: Supplementary file 1 [file materials-16-02442-s001.zip › materials-1748350-supplementary.pdf]

Morphology determines an efficient coherent electron transport for push-pull organic semiconductors based on triphenylamine and dicianovinyl groups

Alexander Romero[1] Jaime Velasco-Medina[1]* and Alejandro Ortiz[2]

1. Grupo de Bionanoelectrónica, Universidad del Valle, Calle 13 # 100-00, Cali, Colombia
2. Grupo de Investigación de Compuestos Heterocíclicos, Universidad del Valle, Calle 13 # 100-00, Cali, Colombia

*Corresponding author
Email address: jaime.velasco@correounivalle.edu.co

Supplementary Information

S1. Geometry Optimization

In Table S1, we reported the number of k-points used for sampling the Brillouin zone during simulations of geometry optimization. For an even number of k-points, the grid was shifted to include the Γ point. In Table S2, we reported the cell geometry before and after geometry optimization. Even when imposing an orthorhombic geometry, the length of the lattice vector does not change substantially, and the stress is around the same magnitude as the obtained without constraining the Bravais lattice type. For compound 3, it was necessary to build a conventional cell (base-centered monoclinic) before transforming the geometry. The above avoids excessive deformation of the molecular geometry.

Table S1. k-points used in geometry optimization

Compound	1		2		3		4	
Reciprocal vector	k-points number	k-points density [Å]	k-points number	k-points density [Å]	k-points number	k-points density [Å]	k-points number	k-points density [Å]
a	3	9.5	9	9.9	2	9.6	11	16.4
b	9	10.3	7	10.2	6	9.4	2	16.2
c	4	9.9	3	9.7	4	10.2	10	16.2

Table S2. Lattice geometry

Compound		1			2			3			4		
Structure*		Exp	Opt	Opt-Ort	Exp	Opt	Opt-Ort	Exp	Opt	Opt-Ort	Exp	Opt	Opt-Ort
Crystal system**		m		o	a		o	m		o	m		o
	a	19.84	19.40	18.80	6.89	6.53	6.64	30.11	29.61	30.31	9.36	9.35	9.39
Lattice vectors lengths [Å]	b	7.19	7.02	7.29	9.195	8.94	9.13	9.89	9.95	9.54	50.98	50.56	51.64
	c	15.61	15.25	14.84	20.41	20.29	19.42	16.04	15.81	15.95	10.15	9.99	9.83
	α	90	90	90	96.35	94.91	90	90	90	90	90	90	90
Lattice angles [°]	β	110.60	109.59	90	95.24	93.74	90	94.18	96.38	90	102.22	102.56	90
	γ	90	90	90	100.90	103.29	90	90	90	90	90	90	90
Stress [GPa]		20.57	0.09	0.09	16.65	0.08	0.09	16.62	0.08	0.07	17.57	0.06	0.05

* Exp=Experimental, Opt=Optimized, Opt-Ort=Optimized forcing the orthorhombic geometry

** m=monoclinic, a= triclinic, o=orthorhombic

S2. Electronic Band Structure

Table S3. k-points used in the calculation of the electronic band structure

Compound		1		2		3		4	
k-points set	Reciprocal vector	k-points number	k-points density [Å]	k-points number	k-points density [Å]	k-points number	k-points density [Å]	k-points number	k-points density [Å]
	a	3	9.0	9	9.5	2	9.6	6	9.0
1	b	7	8.1	7	10.2	6	9.1	1	8.2
	c	3	7.1	3	9.3	4	10.2	5	7.8
	a	5	15.0	15	15.8	3	14.5	11	16.4
2	b	13	15.1	11	16.0	9	13.7	2	16.4
	c	7	16.5	5	15.5	7	17.8	10	15.6
	a	7	20.9	21	22.2	5	24.1	16	23.9
3	b	19	22.0	15	21.8	15	22.8	3	24.7
	c	9	21.3	7	21.6	9	22.8	15	23.5
	a	9	26.9	27	28.5	7	33.8	21	31.4
4	b	25	29.0	19	27.6	21	31.9	4	32.9
	c	11	26.0	9	27.8	13	33.0	19	29.7
	a	17	50.9	49	51.8	11	53.1	37	55.3
5	b	47	54.5	35	50.9	33	50.1	7	57.5
	c	21	49.6	15	46.4	21	53.3	35	54.8
	a	31	92.7						
6	b	79	91.6						
	c	39	92.1						
	a	33	98.7						
7	b	87	100.9						
	c	43	101.6						
	a	35	104.7						
8	b	91	105.5						
	c	45	106.3						
	a	37	110.7						
9	b	95	110.2						
	c	47	111.0						
	a	39	116.7						
10	b	101	117.1						
	c	49	115.7						

Table S4. Variation of the band gap and Fermi level with respect to real and reciprocal space sampling

Compound		1				2				3				4			
k-points set	Mesh cut-off [E_h]	VBE	CBE	BG	FL	VBE	CBE	BG	FL	VBE	CBE	BG	FL	VBE	CBE	BG	FL
	40	-0.67	0.59	1.26	-4.09	-0.49	0.50	1.00	-4.27	-0.45	0.41	0.86	-4.32	-0.49	0.48	0.97	-4.15
	80	-0.67	0.59	1.26	-4.09	-0.49	0.50	1.00	-4.27	-0.45	0.41	0.86	-4.32	-0.49	0.48	0.97	-4.14
1	100	-0.67	0.59	1.26	-4.09	-0.49	0.50	1.00	-4.27	-0.45	0.41	0.86	-4.31	-0.49	0.48	0.97	-4.14
	160	-0.67	0.59	1.26	-4.09	-0.49	0.50	1.00	-4.27	-0.45	0.41	0.86	-4.31	-0.49	0.48	0.97	-4.14
	200	-0.67	0.59	1.26	-4.09	-0.49	0.50	1.00	-4.27	-0.45	0.41	0.86	-4.31	-0.49	0.48	0.97	-4.14
	40	-0.71	0.55	1.26	-4.04	-0.49	0.50	1.00	-4.27	-0.45	0.41	0.86	-4.32	-0.49	0.47	0.97	-4.14
	80	-0.71	0.55	1.26	-4.04	-0.49	0.50	1.00	-4.27	-0.45	0.41	0.86	-4.32	-0.49	0.47	0.97	-4.14
2	100	-0.71	0.55	1.26	-4.04	-0.49	0.50	1.00	-4.27	-0.45	0.41	0.86	-4.31	-0.49	0.47	0.97	-4.14
	160	-0.71	0.55	1.26	-4.04	-0.49	0.50	1.00	-4.27	-0.45	0.41	0.86	-4.31	-0.49	0.47	0.97	-4.14
	200	-0.71	0.55	1.26	-4.04	-0.49	0.50	1.00	-4.27	-0.45	0.41	0.86	-4.31	-0.50	0.47	0.97	-4.14
	40	-0.63	0.63	1.26	-4.13	-0.49	0.50	1.00	-4.28	-0.45	0.41	0.86	-4.32	-0.50	0.47	0.97	-4.14
	80	-0.63	0.63	1.26	-4.12	-0.49	0.50	1.00	-4.27	-0.45	0.41	0.86	-4.32	-0.50	0.47	0.97	-4.14
3	100	-0.63	0.63	1.26	-4.12	-0.49	0.50	1.00	-4.27	-0.45	0.41	0.86	-4.31	-0.50	0.47	0.97	-4.14
	160	-0.63	0.63	1.26	-4.12	-0.49	0.50	1.00	-4.27	-0.45	0.41	0.86	-4.31	-0.50	0.47	0.97	-4.14
	200	-0.63	0.63	1.26	-4.12	-0.49	0.50	1.00	-4.27	-0.45	0.41	0.86	-4.31	-0.50	0.47	0.97	-4.14
	40	-0.75	0.51	1.26	-4.00	-0.50	0.50	1.00	-4.27	-0.45	0.41	0.86	-4.32	-0.50	0.47	0.97	-4.14
	80	-0.75	0.51	1.26	-4.00	-0.50	0.50	1.00	-4.27	-0.45	0.41	0.86	-4.32	-0.50	0.47	0.97	-4.14
4	100	-0.75	0.51	1.26	-4.00	-0.50	0.50	1.00	-4.27	-0.45	0.41	0.86	-4.32	-0.50	0.47	0.97	-4.14
	160	-0.75	0.51	1.26	-4.00	-0.50	0.50	1.00	-4.27	-0.45	0.41	0.86	-4.31	-0.50	0.47	0.97	-4.14
	200	-0.75	0.51	1.26	-4.00	-0.50	0.50	1.00	-4.26	-0.45	0.41	0.86	-4.31	-0.50	0.47	0.97	-4.14
	40	-0.81	0.45	1.26	-3.94	-0.52	0.47	1.00	-4.24	-0.45	0.41	0.86	-4.32	-0.49	0.47	0.97	-4.14
	80	-0.81	0.45	1.26	-3.94	-0.52	0.47	1.00	-4.24	-0.45	0.41	0.86	-4.32	-0.49	0.47	0.97	-4.14
5	100	-0.81	0.45	1.26	-3.94	-0.52	0.47	1.00	-4.24	-0.45	0.41	0.86	-4.32	-0.50	0.47	0.97	-4.14
	160	-0.81	0.45	1.26	-3.94	-0.52	0.47	1.00	-4.24	-0.45	0.41	0.86	-4.32	-0.49	0.47	0.97	-4.14
	200	-0.81	0.45	1.26	-3.94	-0.52	0.47	1.00	-4.24	-0.45	0.41	0.86	-4.32	-0.50	0.47	0.97	-4.14
6	40	-0.86	0.40	1.30	1.26	1.26	-3.90										
7	40	-0.85	0.41	1.30	1.26	1.26	-3.90										
8	40	-0.42	0.84	1.30	1.26	1.26	-4.34										
9	40	-0.82	0.44	1.30	1.26	1.26	-3.94										
10	40	-0.46	0.80	1.30	1.26	1.26	-4.30										

VBE=Valence band edge, CBE=Conduction band edge, BG=Band gap, FL= Fermi level; all these energies are in eV.

In table S4, the values highlighted in red are outliers of the general trend for the Fermi level of compound 1.

S3. Electronic transmission spectrum

Figures S1 and S2 show that the general features of the transmission spectrum are the same when this spectrum is calculated using a low or high k-points density. For compound 1, Figure S2 shows that the transmission spectrum shifts with respect to the Fermi level when the band structure is calculated using a low or high k-points density.

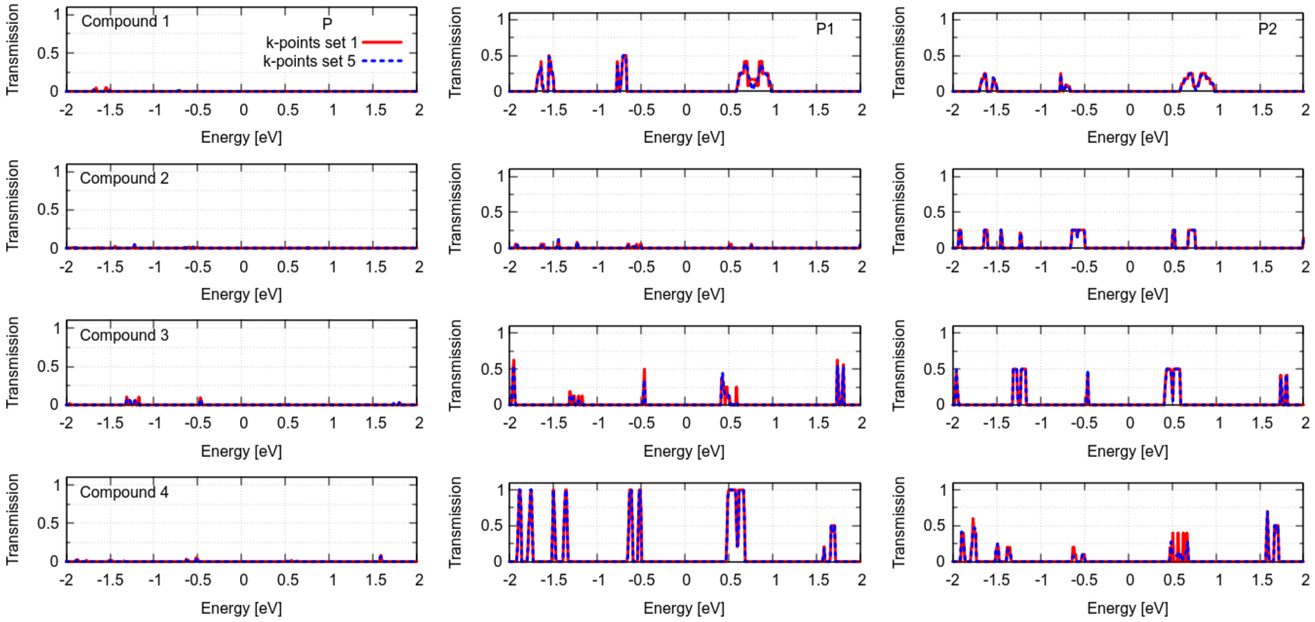

Figure S1. Transmission spectrum calculated using k-points sets 1 and 5, which corresponds to k-points densities of ~9 and 52 Å, respectively. The band structure was calculated using the k-points set 1. The energy is relative to the Fermi level, which is set to zero.

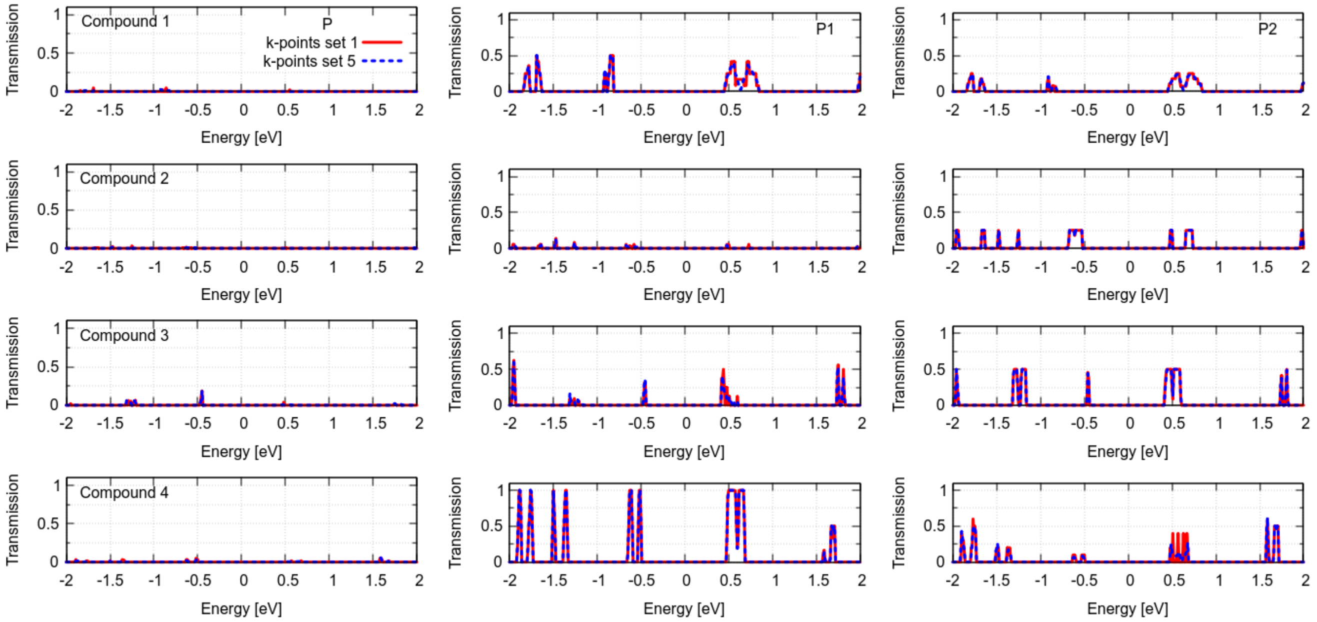

Figure S2. Transmission spectrum calculated using k-points sets 1 and 5, which corresponds to k-points densities of ~9 and 52 Å, respectively. The band structure was calculated using the k-points set 5.The energy is relative to the Fermi level, which is set to zero.

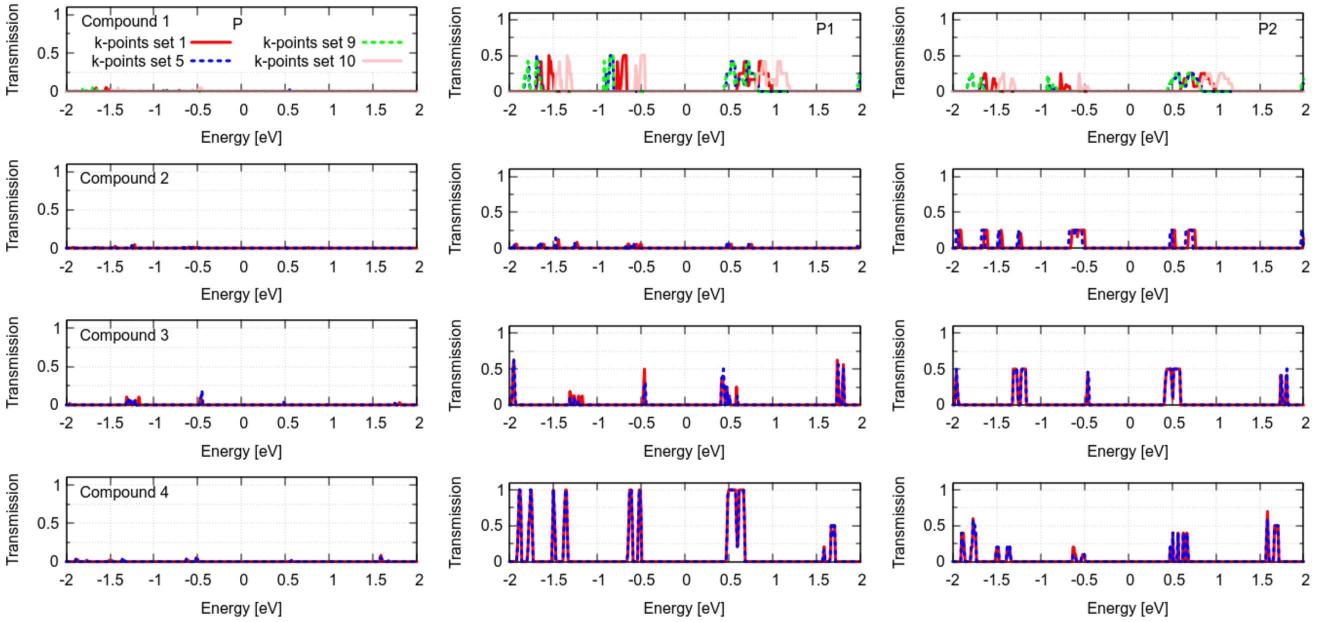

Figure S3. Transmission spectrum calculated using k-points set 1. The band structure was calculated using using k-points sets 1 and 5, which corresponds to k-points densities of ~9 and 52 Å, respectively. The energy is relative to the Fermi level, which is set to zero.
